# Split-based points from the Swabian Jura highlight Aurignacian regional signatures

**DOI:** 10.1371/journal.pone.0239865

**Published:** 2020-11-10

**Authors:** Keiko Kitagawa, Nicholas J. Conard

**Affiliations:** 1 SFB 1070 ResourceCultures, University of Tübingen, Tübingen, Germany; 2 Department of Early Prehistory and Quaternary Ecology, University of Tübingen, Tübingen, Germany; 3 Senckenberg Centre for Human Evolution and Paleoenvironment, University of Tübingen, Tübingen, Germany; University at Buffalo - The State University of New York, UNITED STATES

## Abstract

The systematic use of antlers and other osseous materials by modern humans marks a set of cultural and technological innovations in the early Upper Paleolithic, as is seen most clearly in the Aurignacian. Split-based points, which are one of the most common osseous tools, are present throughout most regions where the Aurignacian is documented. Using results from recent and ongoing excavations at Geißenklösterle, Hohle Fels and Vogelherd, we nearly tripled the sample of split-based points from 31 to 87 specimens, and thereby enhance our understanding of the technological economy surrounding the production of osseous tools. Aurignacian people of the Swabian Jura typically left spit-based points at sites that appear to be base camps rich with numerous examples of personal ornaments, figurative art, symbolic imagery, and musical instruments. The artifact assemblages from SW Germany highlight a production sequence that resembles that of SW France and Cantabria, except for the absence of tongued pieces. Our study documents the life histories of osseous tools and demonstrates templates for manufacture, use, recycling, and discard of these archetypal artifacts from the Aurignacian. The study also underlines the diversified repertoire of modern humans in cultural and technological realms highlighting their adaptive capabilities.

## Introduction

The use of osseous material increased and diversified with the emergence of modern humans in the Old World [1–4]. This is accompanied by the development of complex artifact types and tool systems, a tradition that persisted as modern humans spread into Eurasia [5–8]. In the European Aurignacian, we observe a rich array of osseous artifacts with functional as well as symbolic values, the latter being represented by musical instruments, personal ornaments, and figurative art including therianthropic imagery [9–15].

The Swabian Jura is one of the key regions with rich evidence of bone, antler and ivory working dated to the early Upper Paleolithic (eUP) [9, 10, 12, 16–18]. Prominent cave sites located in the Ach and Lone valleys have yielded a record of the Aurignacian that is crucial to our understanding of early modern humans in Central and greater Europe, which has led to their recognition as a UNESCO World Heritage site in 2017 [19]. The body of osseous artifacts over multiple sites provides an opportunity to consider technological parallels and variability on an inter-regional scale, revealing a more complete view of the Aurignacian technology.

Antler points, specifically split-based points (SBP), count among the most numerous and wide-spread examples of osseous tools from the eUP [20–23]. This innovation represents one of the complex weaponry systems that are in the repertoire of modern humans’ behavioral complexity [24–27]. In Europe, this technological shift also signals the use of a new raw material, antler, which was subsequently used throughout the Upper Paleolithic, underscoring its lasting utility for the Late Pleistocene hunter-gatherers [22, 23, 28]. SBPs also play an important role in Paleolithic study ever since researchers from their discovery in the 1860s have equated their presence with the Aurignacian technocomplex [29].

This paper explores the largest assemblage of SBPs from Central Europe recovered from the Swabian Jura. In doing so, we augment our understanding of the Aurignacian technology, which demonstrated greater reliance on osseous tools than earlier archaeological cultures. This general trend, whose origin may lie in the Middle Stone Age (MSA) cultures of Africa, feeds into the larger discussion of innovations in human behavior and evolution. Ultimately, an in-depth study of osseous technology from eUP fosters more discussion into the question of how osseous raw materials were incorporated into the repertoire of formal tool making and what the diversification of tool systems means for the behavioral complexity in the course of human evolution.

## Background

Along with other osseous tools, the SBP was first documented as a formal tool made from antlers by Lartet in his publication on the site of Aurignac [29]. Breuil later named the SBP an *Aurignac* point and noted its wide-spread distribution due to its presence across multiple sites, suggesting a clear emergence of point typology [30]. In 1911, Didon additionally documented a diverse array of osseous tools including SBPs, which were recovered from Abri Blanchard [31].

Peyrony was the first to propose a chronological model of the Aurignacian with five subphases based on different types of organic projectile point from La Ferrassie and Laugerie-Haute. His work also contributed to the first discussion of the production method [32, 33]. Since his publications, others have followed suit with different chrono-typological schemes, which varied according to the analyzed assemblages and their theoretical approach [34, 35]. This earlier period of the research history was dominated by French scholars as the rich Aurignacian material was concentrated in the Aquitaine Basin of France and the neighboring region of Cantabria in Spain. According to many models which drew from these studies, the SBPs represent the earliest subphase of the Aurignacian (*Aurignacien ancien* or Aurignacian I), which explains the regional chronological trends in the development of technological repertoires.

SBPs were synonymous with the Aurignacian since its initial discovery and its status as an index fossil was established early on [36, 37]. Tafelmaier recently reviewed the distribution and documentation of SBPs Europe wide, highlighting inconsistencies in the chronology of early Aurignacian and their presence as well as the need to integrate better radiometric data to ultimately understand the processes underlying the spread of the Aurignacian culture [38]. The SBP is uniquely ascribed to the early Upper Paleolithic, but the fine details such as when they appeared in multiple regions and what kind of cultural links existed between the Proto-Aurignacian and Early Aurignacian in the Mediterranean region require further investigation [37, 39].

Later studies provide an overview of projectile points from the Upper Paleolithic [40–42]. Albrecht and colleagues [41] studied osseous projectile points spanning from the Middle Paleolithic to the Gravettian in multiple sites across Central and Eastern Europe by investigating basic metric data of the projectile points, noting greater intersite variability than intrasite variability. In addition, other researchers such as Knecht and Hahn expanded their studies by incorporating and comparing assemblages from Central and Western Europe to gain an inter-regional perspective [40, 43]. Recent studies have continued to add more assemblages to the distribution map of Aurignacian sites with SBPs [44, 45], allowing for a more global approach to the analysis of the antler points including morphometrics [46].

SBPs served as projectile tips for hunting weapons. Other uses have been suggested, but largely remain hypothetical. Early on, the SBP was recognized as a composite tool due to the general morphology and the particular feature at the proximal ends [29]. The splits on the base provide evidence of hafting and result in a morphology that is unique to the Aurignacian antler points. eUP makers took advantage of its elastic and durable properties, a substantial shift from the previous projectile technology that largely relied on lithic artifacts [27]. Their dimension also hints at the emergence of weapons, which combine bone and lithic elements as a new form of composite tool [40].

Didon first documented worked antler pieces later known as tongued pieces (*languette*), which were associated with SBPs [31]. Peyrony later linked these finds to the production of the splits on the antler points. His work explained how the hafting end can be produced by incising one side of the proximal part and flexing it to create a fissure which results in the detachment of the tongued pieces from the newly made base with a split [33, 47]. Henri-Martin [48] proposed another method to create a hafting base, which involved the use of cleavage. These competing hypotheses have been explored through multiple experimental studies [44, 49–51]. Knecht also proposed that these tongued pieces had an additional role as a binding piece which aided in the hafting process of SBPs [27]. In some recent experiments, the method of incision and flexion appeared to have a higher success rate than the cleavage method to initiate a split [44]. Currently, two hypotheses on the splitting method exist with no clear consensus.

Massive based points (MBPs) represent another variant of antler projectile points that are recovered from Aurignacian contexts. While the morphology of the proximal end can resemble that of SBPs, MBPs lack the basal split. Furthermore, the raw material of MBP varied from antler, bone to ivory unlike SBP, which was predominantly made from antler with few exceptions. While Henri-Martin distinguished and identified points without splits [52], Leroy-Prost described these points in detail and referred to the points as MBP [53, 54]. In Europe, it appears that these points emerged during the later phases of the Aurignacian (*Aurignacien évolué*) [53] and were more common in Eastern Europe [39]. The extent to which MBP and SBP coexisted needs further exploration, but it appears that both geographical and temporal variations factor into their distribution.

In the Swabian Jura, Bürger’s excavation in 1883–4 of Bockstein-Höhle yielded the first SBP from the region [55]. In 1931, Riek’s excavation of Vogelherd highlighted the richness and diversity of osseous tools, which regionally existed in the Aurignacian culture, including SBPs. Subsequently, these assemblages in addition to others were studied by Albrecht, Torke, and Hahn for a comparative overview (1972). Hahn also conducted a survey of the SBP from the Swabian Jura and SW France, highlighting a broader pattern of the morphological variation (1988) and similar work was conducted by Knecht (1990) [56]. Liolios [49] closely investigated and compared osseous assemblages from two areas, Swabian Jura and SW France. A recent study of the projectile points explored the chronological trends and different types of armature technology from the eUP, demonstrating the variability of projectile technology over time [57].

## Swabian Jura

The Swabian Jura represents the largest karstic region of Jurassic limestones in southwestern Germany and is part of a range of the Jura mountains which stretch across Germany, Switzerland, and France. The topography is characterized by a plateau with altitudes ranging between 450–1000 m asl. The area is bound by the Upper Danube Valley that cuts through the Alpine Foreland to the south. The current river drainage system formed during the Riss Glacial period (200–130 ka), which affected the courses of the Danube and its tributaries with the advances of the glaciers in the Alps [58–60].

**Fig 1:**
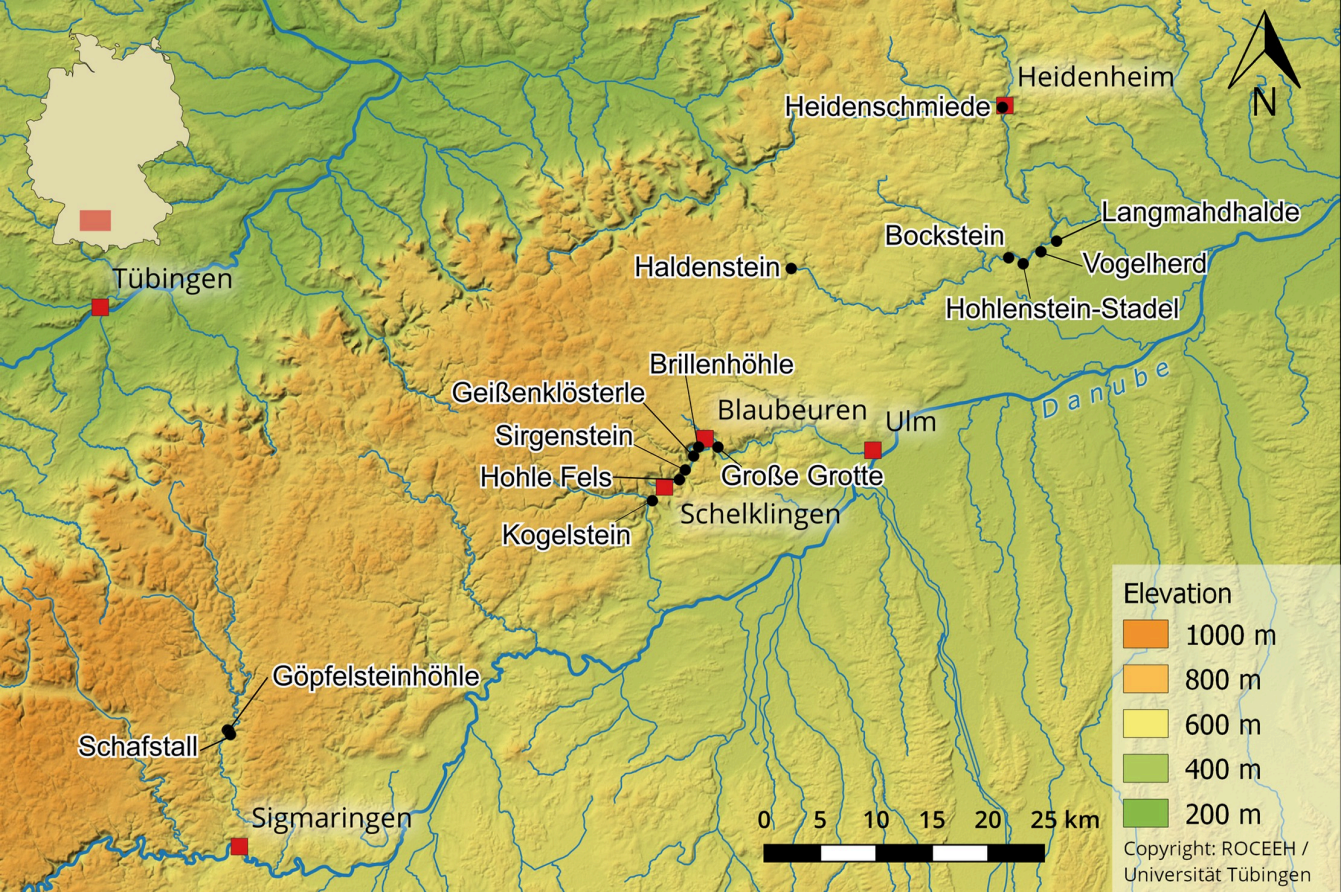
Map of the major Paleolithic sites in the Swabian Jura. Lone Valley: Vogelherd; Hohlenstein-Stadel; Bockstein; 5. Haldenstein. Ach Valley: Große Grotte; Brillenhöhle; Geißenklösterle; Sirgenstein; Hohle Fels; Kogelstein. Lauchert Valley: Schafstall; Göpfelsteinhöhle. Brenz Valley: Heidenschmiede (https://doi.org/10.5281/zenodo.3460301) © ROCEEH / University of Tübingen.

The majority of important Aurignacian deposits are located in the Ach and Lone valleys, which are ~15–30 km apart (Fig 1). We also refer to one osseous tool from Schafstall II [61], which is located in the Lauchert Valley, southwest of the Ach and Lone valleys. Archeological sites are either caves or rockshelters with little evidence of open-air occupation (but see Bolus, Conard [62], Floss, Fröhle [63]).

The Aurignacian deposits in the Swabian Jura have produced one of the rich records which are dated to the eUP. The material culture exhibits a rich array of lithic tools as well as osseous artifacts, showing clear intensification in the site use compared to the Middle Paleolithic settlements [64–67]. The record has also yielded rich and unique examples of personal ornaments, symbolic imagery, musical instruments and figurative art unique to the region [9, 10, 12, 17]. The medium of most symbolic representations is osseous in nature, ranging from bone, antler and ivory. While comparable osseous artifacts are recovered from other Aurignacian cultural areas, the extensive and diverse exploitation of ivory remains unique in most of the eUP of Europe.

During the MIS 3 (60–20 ka) of the Late Pleistocene, the region experienced continuous climatic fluctuations between the stadial and interstadial periods. Pollen record from Bergsee (47°34’20” N 7°56’11” E) in the Black Forest spanning between 45–30 k cal BP shows that the landscape was characterized by the dominance of Poaceae (40% on average) as well as cyclical development and waning of boreal forests, which included *Juniperus*, *Betula*, and *Pinus* [68]. This corresponds well to the climatic record that was reconstructed from the floral material excavated at the local caves [69].

Additional reconstruction based on the microfauna also suggests that the onset of the Aurignacian is characterized by a mild climate, which is followed by a cooler and drier condition, a pattern reflected in the abundance of two species of lemmings and narrow-headed vole [70–72]. The middle to upper horizons of the Aurignacian saw a return to a mild climate with open boreal elements. Micromorphological studies also reveal fluctuating climates from a mild to cooler condition after the initial onset of the early Upper Paleolithic [59, 73].

The paleoenvironmental record of the early Upper Paleolithic correlates with the increase of reindeer across multiple sites in the Swabian Jura relative to the late Middle Paleolithic [74, 75]. Reindeer were one of the most common prey besides horses and became heavily incorporated into the economic activities of the Aurignacian. Others have documented this trend elsewhere and this appears to be an interregional pattern largely driven by the cooler and drier climate in the Aurignacian in Europe [76]. Other cervids including red deer are present in the faunal record but are rare relative to reindeer.

## Materials and methods

The technological study here is informed by the *chaîne opératoire* approach [77]. The *chaîne opératoire* as defined by the French school engages in a detailed reconstruction of successive stages and organizational system employed in the production of tools, which stems from a structuralist perspective that aligns with Leroi-Gourhan’s theory [77, 78]. A simple iteration of this approach emphasizes the life history of artifacts through production, use, and discard. The ultimate aim is to reconstruct the operational sequence (i.e., steps involved in the creation and use of artifacts) as well as makers’ technique, knowledge, and gestures. The focus on the process underlying the manufacture of objects helps us interpret assemblages from a comprehensive perspective and move away from a simple interpretation of artifacts as static objects. In the last decades, a concerted effort among researchers of the osseous industry has led to exchanges and publications that serve to define terms and foster discussion with a common theoretical and methodological ground [79, 80]. For this paper, we incorporate *chaîne opératoire* by outlining the production sequence and interpreting the body of antler artifacts in an integrated framework. In addition, major morphological features are measured and compared following examples of Albrecht, Hahn, and Knecht (for detailed study in morphometrics see Doyon 2019 [46]).

The eUP material of the Swabian Jura represents the richest archaeological record in Central Europe. Key assemblages include those from Geißenklösterle [66, 67], Hohle Fels [10, 81, 82], Brillenhöhle [83, 84], and Sirgenstein [85] in the Ach Valley in addition to Vogelherd [86], Bockstein [55, 87], and Hohlenstein-Stadel [88] in the Lone Valley. A single site in the Lauchert Valley, Schafstall II, has also produced one relevant find [60, 89]. Assemblages from Geißenklösterle, Hohle Fels, and Vogelherd comprise the majority of the osseous tools studied in this paper. Importantly, Geißenklösterle and Hohle Fels provide detailed documentation of the stratigraphic context of the osseous artifacts under study. No permits were required for the described study, which complied with all relevant regulations.

Hohle Fels is the largest cave in the region and was initially explored by Fraas and Hartmann in 1870–2. Systematic excavation run by the University of Tübingen began in 1977 initially under Hahn’s and continues until the present under Conard’s direction. The stratigraphic study of the main excavation area reveals a slight downslope movement of sediment from the main chamber of the cave [64]. The dates from lower Aurignacian horizons (Va-Vb) range between 41.7–39 k cal BP, while the upper Aurignacian horizons (AH IIIa-IV) range between 39–36 k cal BP [64].

Geißenklösterle is one of the few sites discovered after WWII in 1958. This allowed for a complete exploration of the site with modern recovery techniques [67]. The initial excavation took under Hahn’s guidance and Conard later revisited the site between 2001–2002. The site is currently a rockshelter but it was likely a cave with a chamber during most of the Late Pleistocene and underwent roof collapse. The site lies close to Hohle Fels albeit at a higher altitude, which is reflected by the faunal spectrum [75]. The radiometric dates of the lower Aurignacian horizons (III-IId) range between 43.4–41.9 k cal BP while upper horizons (IIb, IIa, IIn) range between 41.5–39.4 k cal BP and 39.5–36.9 k cal BP [90].

Vogelherd in the Lone Valley served as a base camp that has produced the richest Aurignacian assemblages in Central Europe. The excavation in 1931 under Riek identified two sublayers IV (*Oberes Aurignacien*) and V (*Mitteleres Aurignacien*) [86]. Except for one profile where horizon VI (*Unteres Aurignacien*) exists, horizon V (*Mitteleres Aurignacien*) represents the lowest horizon of the early Upper Paleolithic at this site. Horizon VI (*Unteres Aurignacien*) has been recently reinterpreted as a late Middle Paleolithic layer [91] and appears to be localized. To make the distinction clear, V is referred to as the lower Aurignacian horizon and IV is referred to as the upper Aurignacian horizon in this paper. Radiometric dates show considerable overlap between the IV and V and their temporal range lies between 42.9 to 31.2 k cal BP [92, 93]. Conard’s excavation of Riek’s backdirt between 2005–12 has significantly contributed to the richness of organic artifacts albeit without stratigraphic contexts [94–96].

Bockstein complex was first excavated in 1883–4 by Bürger and Wetzel later expanded the studied area into different niches after WWII. Both Bockstein-Höhle and Bockstein-Törle (VII) have both produced osseous tools from the Aurignacian layers. A date of Bockstein-Törle (VII) chronologically places the layer between 34.7–33.8 k cal BP [93]. No radiometric data are available for Bockstein-Höhle but the artifacts likely derive from layer V.

The main excavation of Hohlenstein-Stadel took place under Wetzel from 1935–9. A recent study under Kind’s direction shows that three sublayers of the Aurignacian existed, all dating between 41.1–39.4 and 36.9–35.6 k cal BP [17, 93]. The deepest horizon has the lowest artifact density.

Brillenhöhle was first excavated by Riek in 1955–1963 (1958) represented by several cultural layers spanning from the Aurignacian to the Magdalenian. SBPs of Brillenhöhle recovered from horizon XIV were one of the few direct dates, which ranged between 36.9–34.5 k cal BP [91]. Sirgenstein was investigated by Schmidt in 1906 and has produced osseous tools from layer V [97, 98]. In the Lauchert Valley, the only clear Aurignacian occupation derives from Schafstall II, which was excavated by Peters beginning in 1935 [61, 89]. Dating of the stratigraphic sequence from Schafstall II is currently in progress.

All SBPs documented in the Swabian Jura are manufactured using antlers. When the pieces are large enough that the general morphology or the cross-section of the beam can be identified, the majority of the antlers recovered from the Aurignacian derive from reindeer [74, 75]. Their abundance can be accounted for by an increase of this cervid species during the Aurignacian and due to the frequent occurrence of females which bear antlers in addition to males. Thus, we assume that based on their abundance and the structural morphology with a thick cortical zone, the Aurignacian groups procured the majority of raw material for antler artifacts from reindeer. When bases are preserved, shed antlers appear to be more common than those which resulted from hunting as faunal assemblages rarely have produced antlers attached to cranial remains [74, 75]. Thus, while the Aurignacian people regularly hunted reindeer, gathering activities likely account for the main acquisition of antlers for tool-making.

The analyzed specimens are housed in the following institutions: Schloss Sammlung, University of Tübingen (Tübingen), Museum of the University of Tübingen (Tübingen), Landesmuseum Württemberg Stuttgart), Urgeschichtliches Museum (Blaubauren), and Ulmer Museum (Ulm). Access to the materials housed in Landesmuseum Württemberg and Ulmer Museum was provided upon request. The rest of the material under study is housed at the University of Tübingen and the Urgeschichtliches Museum, for which no permission is required.

## Results

### Overview and stratigraphic context

The identification of SBP requires that the split on the lateral sides is recognized or that the proximal end is preserved. In addition to 87 SBP pieces, two categories of antler points are presented in Table 1. The first category refers to the distal and mesial parts of antler fragments without any visible split (N = 76). The second category refers to preforms, which were discarded before the base was modified (N = 27).

**Table 1:** The number of antler points and antler artifacts related to the point production.

	cultural layers (AH)	SBP total					Antler preforms	Antler points (distal/mesial)	Antler blanks	Antler byproduct	MBP
			SBP complete/almost complete	SBP distal/distal-mesial	SBP mesial / mesial-proximal	SBP proximal end (wing)					
Bockstein	Törle VII						2				
	Höhle V	1	1				1				
Brillenhöhle	XIV	1	1				1				
Geißenklösterle	IIa	2			2				2	1	
	IIb	8	4	1	1	2		2	3	6	
	Iic	1	1								
	Iid							1	1	2	
	Iin									2	
	III / IIIa							1		3	
Hohle Fels	Iie								1		
	III / IIIa									3	
	IV	4		1	2	1	1	1	2	4	
	Va or Vaa	2				2	1			7	
	Vb	1	1							1	
	IVwf / Vabwf / IV-VI	2	1		1			1		1	
Hohlenstein-Stadel	IV						1				1 poss
Sirgenstein	V						1 poss	3			1 poss
Vogelherd	IV	1				1	1			1	
	V	14	10	4			2	5	4	3	1 poss
	Not Available	49	14	15	12	8	(16)	(62)	(3)	(3)	
	VI?										1 poss
Schafstall II	AUR	1			1						
	Total	87	33	21	19	14	10 (27)	14 (76)	13	34	4 poss

Note 1: At least three other SBP have been documented in Hahn in 1988 not included in the study. Note 2: Vogelherd's preform/distal points/byproducts are in parenthesis as they are not securely associated with the Aurignacian. Note 3: HST antler point is likely 2 separate artifacts.

Seven out of nine sites with Aurignacian deposits in the Swabian Jura have produced SBPs. At the remaining two sites, antler points which are possible MBPs or preforms of SBP were recovered. Vogelherd has yielded the largest set of SBPs, followed by Geißenklösterle and Hohle Fels. This holds not just for SBPs but additionally antler points without clear splits as well as preforms of antler points.

To date, four potential MBPs made from antler have been identified. They include one piece from Hohlenstein-Stadel and Sirgenstein and two from Vogelherd (one is shown in Fig 2.6). One antler point shows a flattened morphology with no evidence of a split has been recovered from horizon VI of Vogelherd [91]. The artifact from Hohlenstein-Stadel is a polished antler piece, which is modified on all faces. While it has a massive end, the morphology of the artifact does not rule out other tool types such as a smoother and its width (35.9 mm) is beyond the size range of MBPs from other regions [100]. At Sirgenstein, one piece shows a tapered end and is the strongest candidate as a MBP (Fig 3). These possible MBPs do not overlap in their dimension or shape with one another. While the distal fragments and preforms can be assigned to MBP, the rarity of complete points without splits justifies ascribing most remains of antler points to SBPs. In all, SBP outnumbers MBP in the Swabian Jura during the earlier and later phases of Aurignacian.

**Fig 2:**
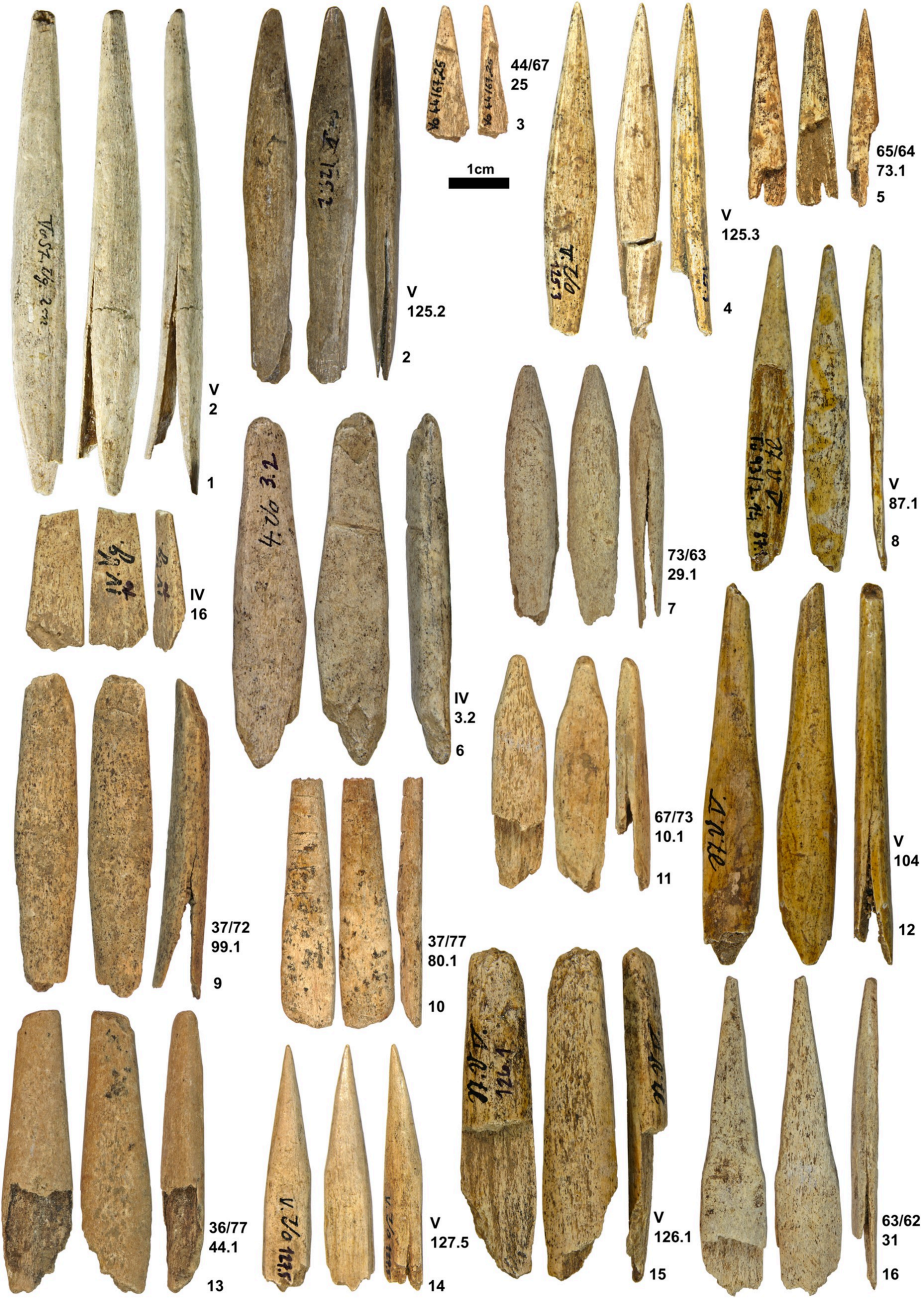
SBPs and point fragments from Vogelherd (IV, V or N/A). Photos taken by K. Kitagawa.

**Fig 3:**
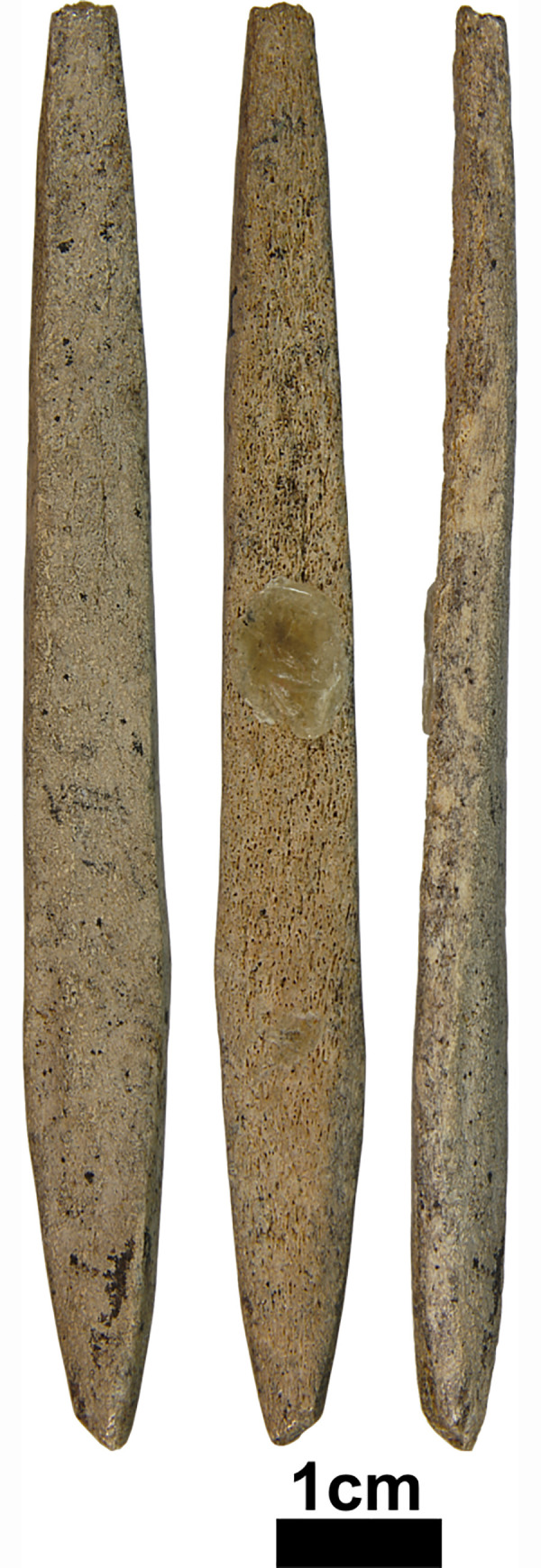
One possible MBP fragment from Sirgenstein (V). Photos taken by K. Kitagawa.

**Fig 4:**
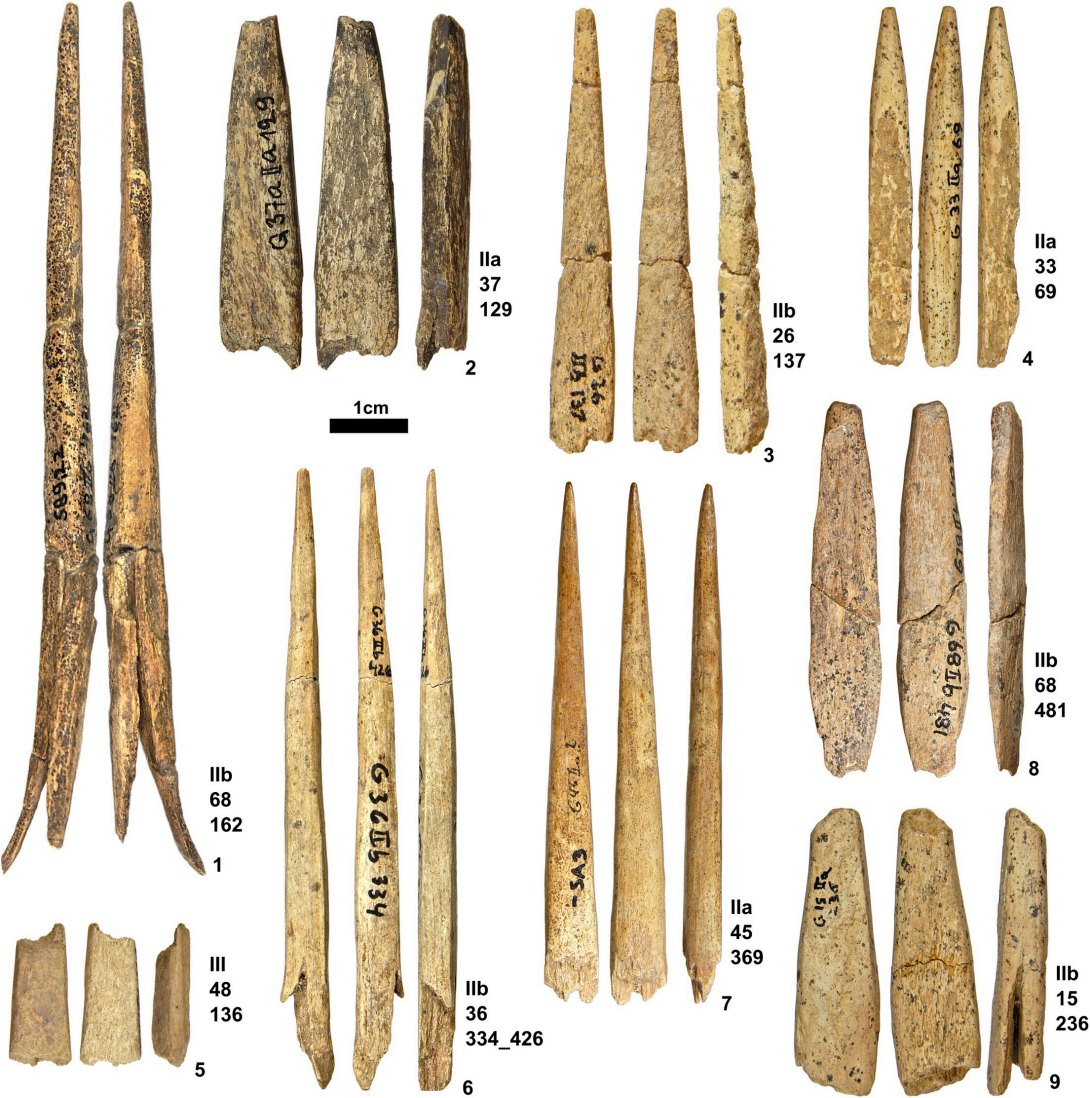
SBPs and point fragments from Geißenklösterle. Photos taken by K. Kitagawa. (Note: 4.1 is housed at the Landesmuseum Württemberg, Stuttgart).

Stratigraphic contexts of SBPs show intersite variability. Geißenklösterle yielded SBPs from the upper Aurignacian layers (IIb, IIa and lln) and not from the lower Aurignacian layers (III or IId) [66] (Fig 4). There is a distal-mesial fragment of an antler point from horizon III (Fig 4.5), but no split is present and no other point with a proximal end has been documented from this horizon. On the other hand, one complete SBP is present from Hohle Fels in layer Vb, which is the basal Aurignacian layer (Fig 5.1) where the female figurine was recovered [10]. While other pieces occur in the upper layers of the Aurignacian (VI, Va, and Vaa) (Figs 4, 5.2 and 6–10), one SBP is associated with one of the earliest presences of Aurignacian cultures in horizon Vb of Hohle Fels.

**Fig 5:**
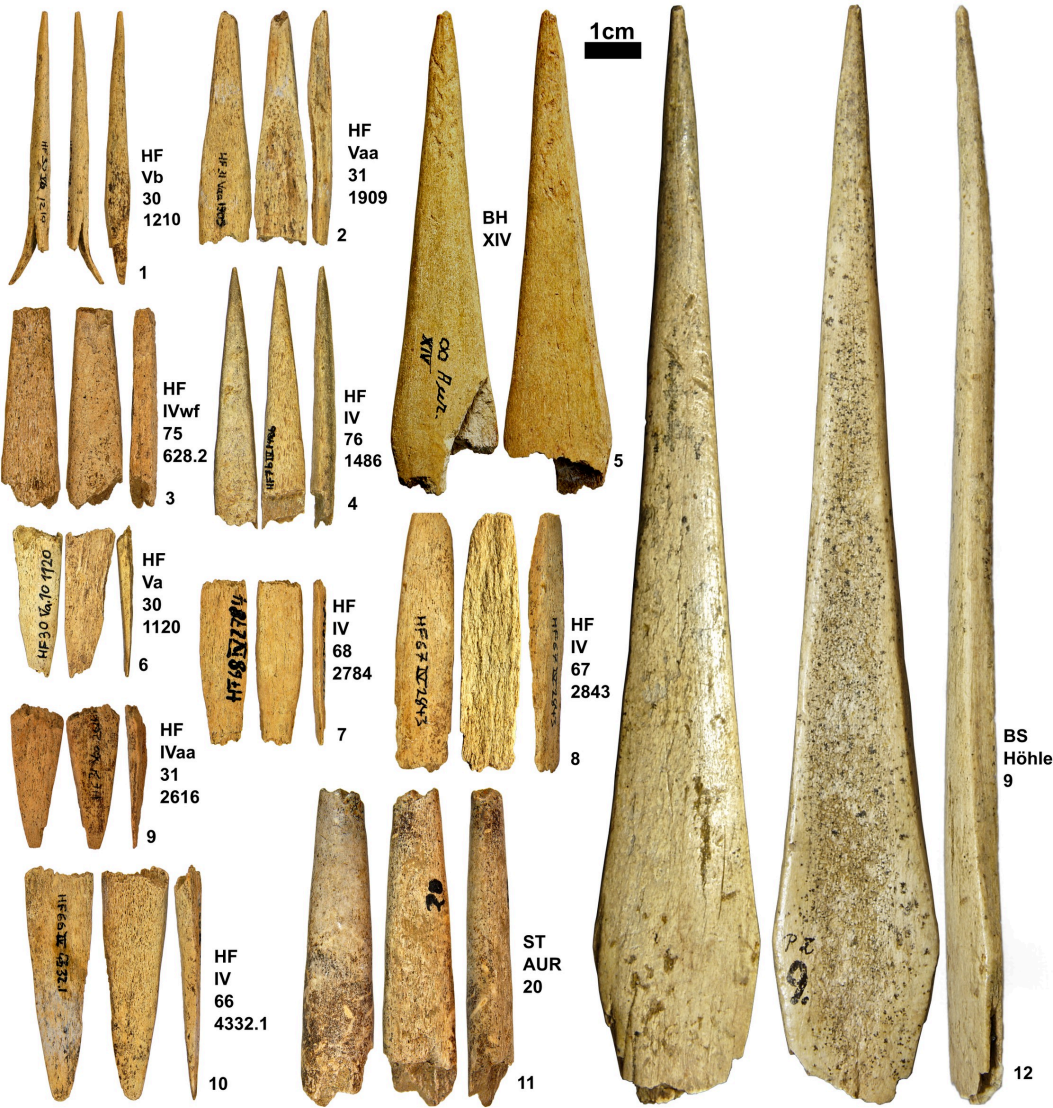
SBPs from Hohle Fels (1–4, 6–11) Brillenhöhle (5) Bockstein-Höhle (12) Photos taken by K. Kitagawa. (5.5 is housed at the Landesmuseum Württemberg, Stuttgart).

**Fig 6:**
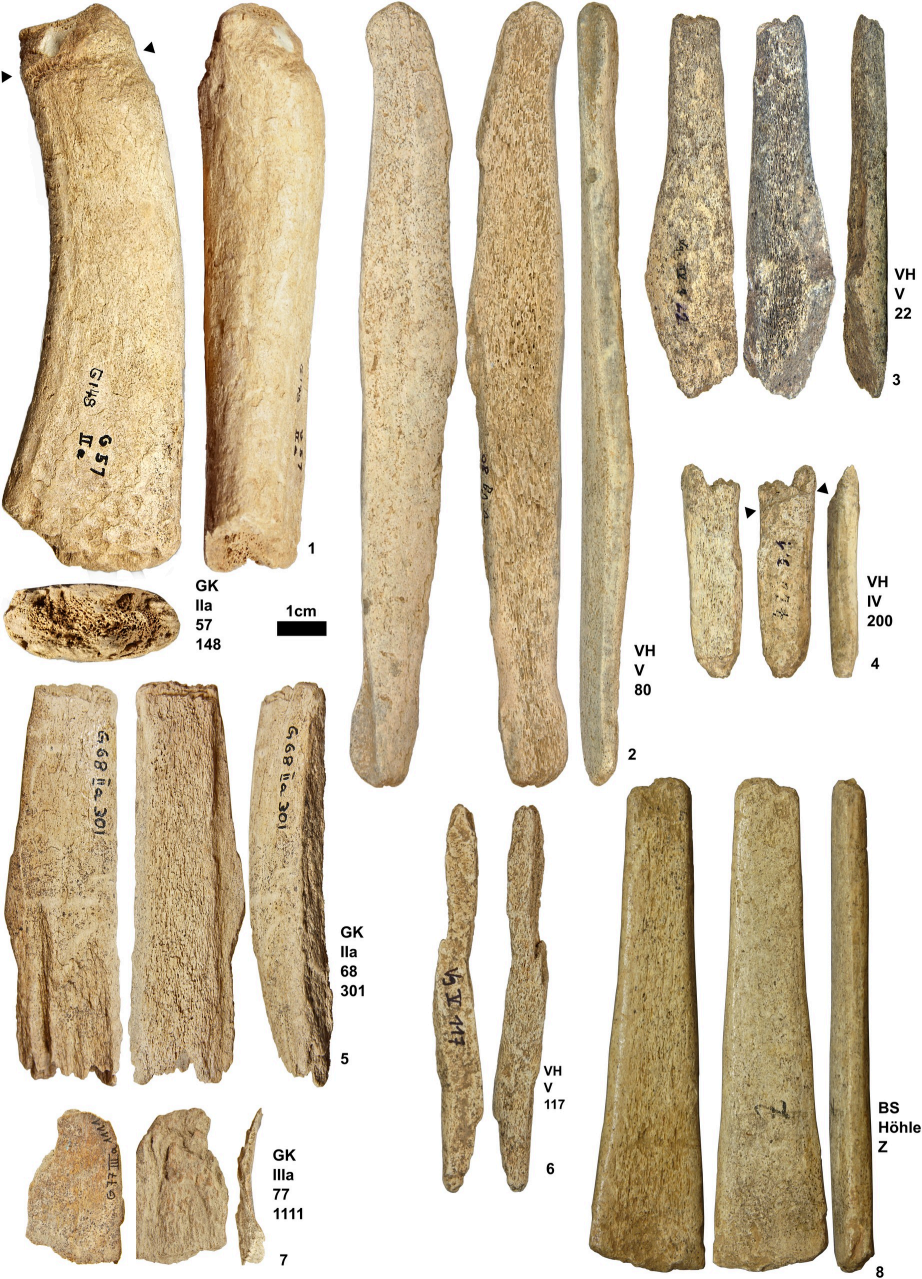
1. Beam section of Geißenklösterle (IIa 57/148). 2 Blank of Vogelherd (V 80). 3 Preform of Vogelherd (IV 22). 4 Byproduct with incision of Vogelherd (IV 200). 5 Blank of Geißenklösterle (IIa 68/301). 6 Byproduct of Vogelherd (V 117). 7 Byproduct of Geißenklösterle (IIIa 77/1111). 8 Preform of Hohle Fels (IV 99/1938). Photos taken by K. Kitagawa.

**Fig 7:**
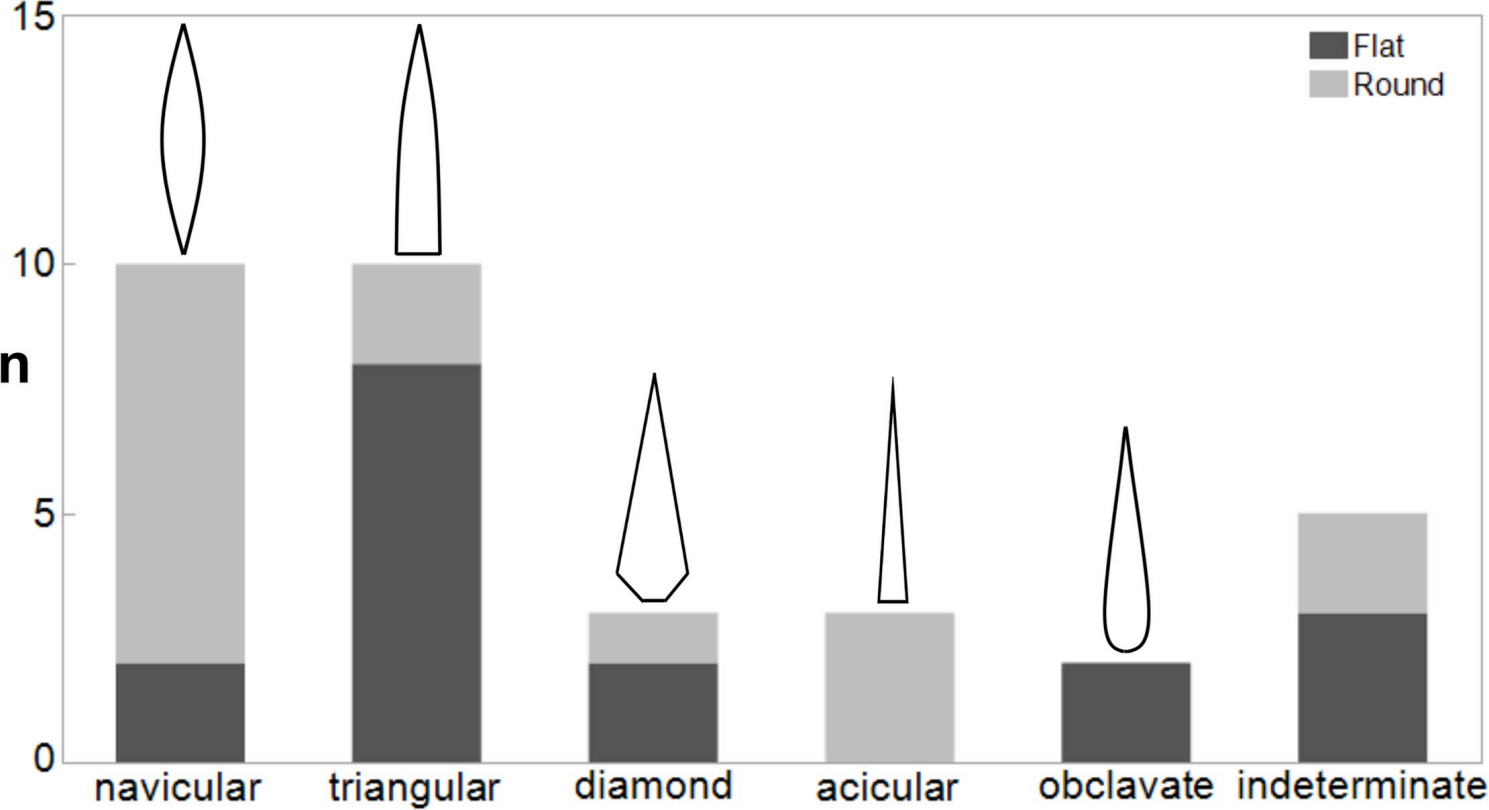
Forms of SBPs (N = 33). Light grey represents flat (>1.5 flatness index) and dark grey represents round pieces (<1.5 flatness index).

**Fig 8:**
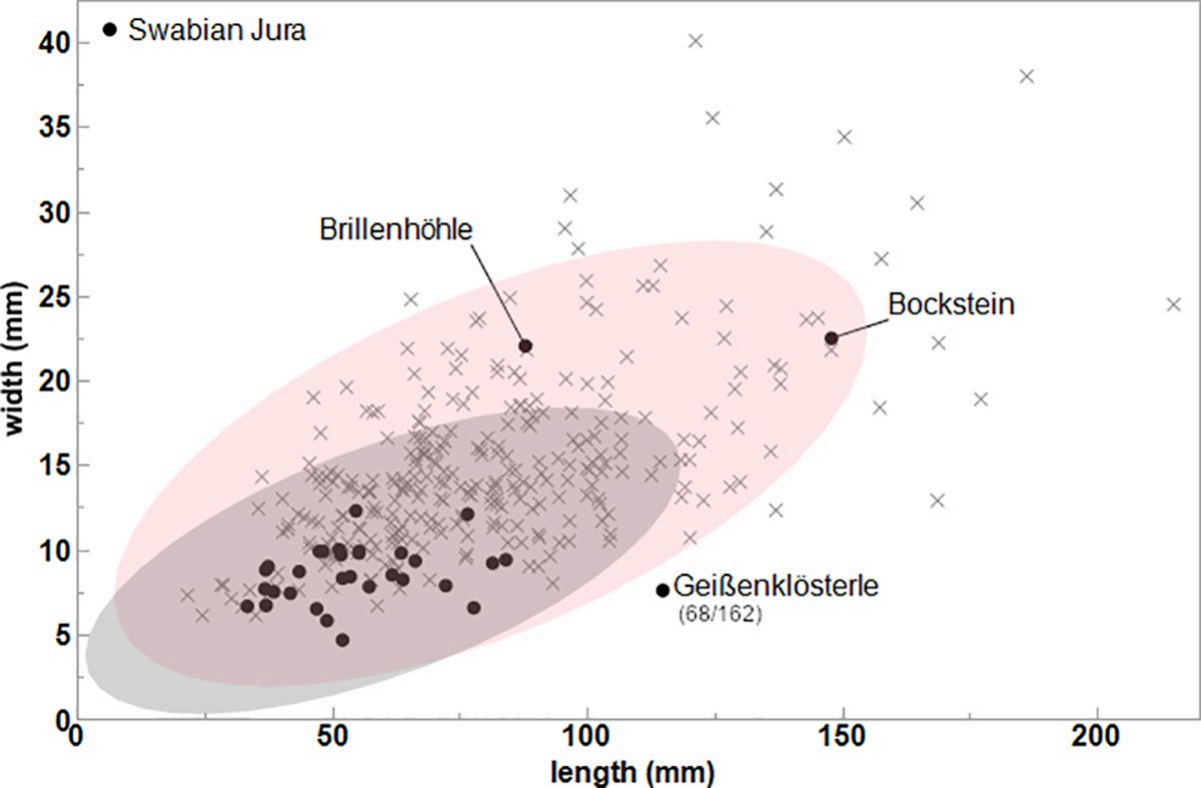
SBPs’ length and width (N = 302) from Europe (Spain, France and Belgium) and SBPs (N = 33) from the Swabian Jura represented by the cross with 95% confidence interval of standard deviations (other measurements from [100]). One piece (Geißenklösterle 68/162) lies outside of the confidence interval of the SBP dimension relative to other European specimens.

**Fig 9:**
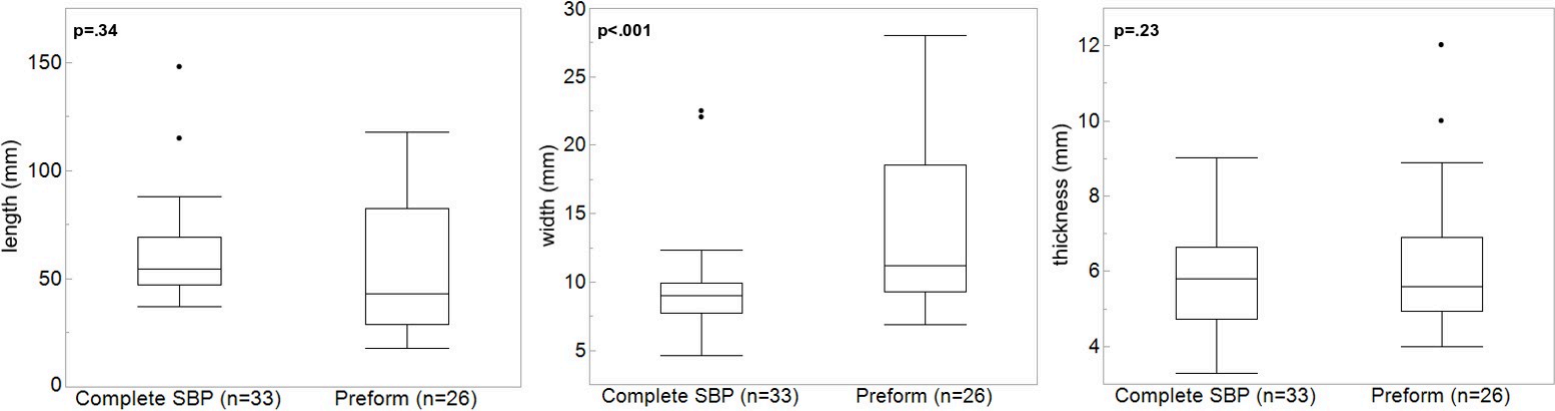
Length, width and thickness distribution of complete SBP preform and complete. The difference of width was statistically significant (p < .05) while others are not.

**Fig 10:**
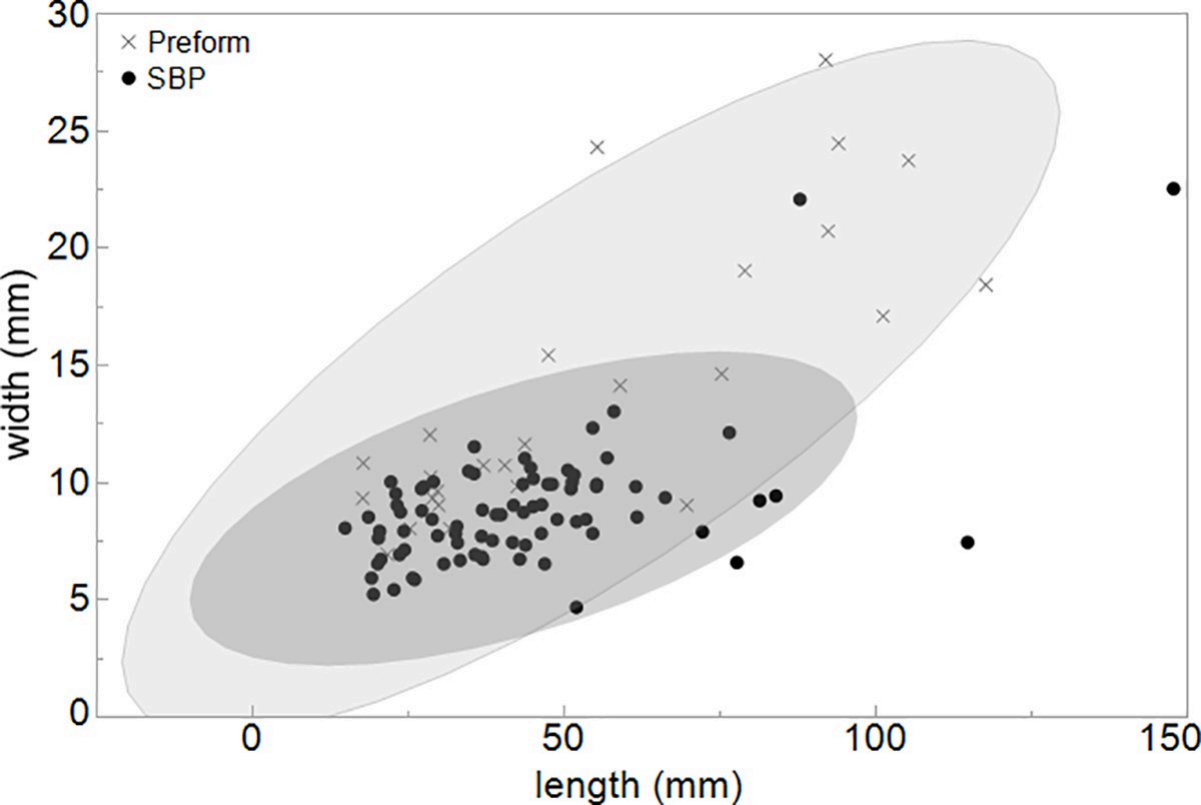
Length and width of all SBPs and preforms with 95% confidence interval.

Likewise, Vogelherd has produced one SBP with a split from the upper Aurignacian (IV) and fourteen SBPs from the lower Aurignacian (V) from the excavation of Riek (Fig 2). Additional samples, comprising more than half of the recovered SBPs at the site, derive either from the early excavation with unclear documentation or recent recovery from Riek’s backdirt which lacks stratigraphic contexts. Direct dates on two SBP pieces from the unstratified contexts align with other dates produced from Vogelherd, and date to the later Aurignacian (OxA-24157: 32,350±450 BP [35,986–37,759 calBP] and OxA-24158: 31,150±400 BP [34746–35646 calBP]).

### Operational sequence

Additional antler remains reveal the operational sequence that is involved in the production of antler points similar to other regions [49]. We observed modified antler pieces that were documented in their experimental studies [44, 45]. For the first step, a relatively complete section of an antler beam, otherwise known as a secondary block [99] is extracted from a complete antler likely with the use of hammerstones and intermediary tools (i.e. wedge). One example shows modification on one end with a circular cross-section and fresh break on the other, whose form is close to an oval shape (Fig 6.1). The marks on one end circle around the entire section. The removal scars resulting from nicking or indirect percussion extend 1.5 cm from the actual end, resulting in a beveled form. The fresh break on the oval end with a jagged fracture corresponds to the techniques of incision and flexion [99]. This piece bear traces of the initial sectioning of the antler beam.

Next, the extraction of blanks follows through a longitudinal splitting of an antler section. Blanks from Vogelherd and Geißenklösterle show a rectangular form with parallel lateral edges, also known as lateral fracture planes. Some pieces are blanks with convergent edges, which occur alongside those with a rectangular form (Fig 6.2). The end of this piece is triangular-shaped and the lateral edge of the blank shows scraping marks that resulted in the rounding of this margin. This piece is one of the few blanks that show modification, which reveals its transition into a preform.

Importantly, the edges of the blanks indicate a possible variation in the extraction method. One blank shows a percussion notch, revealing that one-point direct percussion was applied tangentially on the cortical surface of the antler piece. Another piece shows a series of small notch negatives aligned along with one of the lateral edges, resulting in a slightly sinuous morphology of the fracture plane. This indicates a series of indirect percussion perpendicular to the length of the section suggesting the use of a wedge.

The production of preforms involves the creation of the distal ends by shaping convergent edges in a pointed form (Fig 6.3). Working of preforms also involves near to complete removal of spongy material from the inner surface so that the thickness of the point is consistent across the long axis (Fig 6.8). At this stage, makers work on all cortical, inner, and lateral sides as shown by the inner faces that bear traces of modification. Some pieces also show modified surfaces mostly in the form of scraping marks, which run along the axis of the preform. These working traces are often visible on the pieces when the action was applied to the cortical surface, although some artifacts only show changes in the general morphology.

This is followed by splitting. To date, no artifacts bear clear traces that help reconstruct the splitting technique with the incision and flexion method. A piece shows a possible incision that is oblique to the long axis of a piece on the cortical side with no clear modification on the inner face as spongiosa are still present (Fig 6.4). Due to the presence of the cancellous part, it is unlikely that it served as the incision for a split unless this step occurred before reducing the piece to its cortical structure. It is, in any case, a byproduct resulting from the extraction of the ends either from a blank or preform, but there is no evidence of artifacts with clear tongued morphology and production wear on both inner and cortical surfaces. An incision is also visible on a different worked antler artifact (Fig 2.6). This point shows fresh breaks on the distal and the proximal ends, while the incision is placed in the mesial area. If the incisions were intentional, then the piece would represent a preform which broke during its manufacture and shows the maker’s attempt to rejuvenate the artifact.

The final step involves shaping and smoothing of the general surface from all directions, resulting in a general symmetry of the overall form. The final sharpening of the tip, which is the most fragile part of the point, also belongs to a stage before the completion of the SBP. This process took place shortly after the working of the proximal end as we have no example of SBP with unfinished distal tip. Some of the finished products reveal longitudinal scraping marks along the long axis of the point (Fig 2.4). However, striations resulting from this abrasive action are not present on all SBPs.

The form of byproducts varies depending on the stage at which they were discarded. Tines with a fresh break are one example of a byproduct associated with pruning that facilitates access to the main beam of the antler at the initial phase. Also, parts of the beam with an intentional fracture on one side are possible cores, which were discarded after the extraction of the secondary block. Several antler fragments were identified as byproducts. One is a fragment of antler beam with intact cancellous material and a cortical part with a fresh break. Some artifacts are elongated fragments from the cortical part of the antler with little cancellous material (Fig 6.6). Lastly, thin flakes (Fig 6.7) from Geißenklösterle were partially detached from the cortical part of the antlers. They can result in any stage from the initial sectioning to the production of blanks.

### Variation in size and form

Out of 87 SBPs, 33 are complete or almost complete in shape (S1 File). The rest of the SBPs (N = 54) show a split break visible from the lateral profile or are proximal wings (N = 14). The proximal wings result from the split, always exposing the rough texture of the inner cortical surface (Figs 2.5, 5.6, 7, 9 and 10). This rough texture indicates a tear that resulted from a force applied through the long axis of the object. Some twenty-seven pieces bear ends with fresh fractures in the distal, mesial, or proximal section, affecting the overall morphology.

We can distinguish five forms of complete or near-complete SBPs (Fig 7).

1. Navicular refers to a torpedo-shaped form. The widest part lies in the mesial area and the edges taper off on both distal and proximal end. (i.e. Figs 2.2, 2.4 and 3.8).
2. Triangular (lanceolate) refers to a form with straight or convex lateral edges converging at the distal end. The widest part lies at the base. (i.e. Figs 2.16 and 4.11).
3. Subulate refers to an awl-like shape, which is similar to triangular but its cross-section is round.
4. Examples of this shape are recovered from Geißenklösterle (Fig 4.1) and Hohle Fels (Fig 5.1).
  Diamond (lozenge) refers to a diamond-like shape with truncation. It is marked by obtuse angles which protrude laterally near the proximal end and this discontinuity of the lateral edges marks the beginning of the split. The SBPs from Bockstein-Höhle (Fig 5.12) and Brillenhöhle are an example of this form. The split of the Bockstein-Höhle piece begins at the discontinuous point of the lateral edges, 22.5 mm above the base. (as well as Fig 4.5)
5. Obclavate refers to a club-like shape with curved lateral edges. The widest part lies in the lower mesial area instead of the base or at the midpoint of the longitudinal axis (Fig 2.12).

In the Swabian assemblages, navicular and triangular forms are more frequent than other forms. Additionally, the flatness index expressed by maximum width/thickness can be used to categorize a point as flat or round. With a cutoff line of 1.5, the SBPs fall evenly into these two categories.

For the pieces which are complete or mostly complete in its form (N = 33), the mean of length is **61.1** mm, ranging between **37–147.9** mm. There is a normal distribution of length with most falling in the range of **43.6–57.3** mm (N = **17**). The maximum width shows variation between **4.7–22.5** mm with a mean of **9.5** mm. The thickness is the most constrained variable, ranging between **3.3–9** mm with a mean of **5.7** mm. We observe that the length and width show a correlation of .**61**. Three pieces lie outside of the 95% confidence interval and are interpreted as outliers: the unique piece from Bockstein (Fig 5.12) and Brillenhöhle (Fig 5.5) as well as one SBP from Geißenklösterle (Fig 4.1). The SBP of Bockstein is longer than other pieces, while Geißenklösterle and Brillenhöhle are also outliers due to both variables.

When we compare the variability described above to complete and fragmentary SBPs from 35 sites in Europe (N = 302), we see that the majority of the complete SBPs fall within the 95% confidence interval of the standard deviations [100] (Fig 8). One exception is the SBP of Geißenklösterle (Fig 4.1), making this piece unique in its morphology and dimension. The correlation between length and width of all SBPs is low, only accounting for 37.6% of the variance, a value that is comparable to the points of the Swabian Jura. Compared to other sites, complete SBPs from the Swabian Jura are relatively small in all dimensions (length/width/thickness), which is statistically significant (p>0.05).

Preforms (N = 26) are characterized by a triangular form with straight lateral edges when they are close to completion (S2 File). Some pieces such as those from Bockstein-Höhle and Bockstein-Törle exhibit a similar shape and dimension. Nine pieces show a fresh break on the mesial part or proximal ends. The dimensions of complete SBP and preforms are largely comparable. We observe that the size of the preforms is 4.7 mm wider on average than complete SBPs, which is statistically significant (p < .05) (Fig 9). However, the length and thickness are not significantly different compared to the complete SBPs. In addition, when we account for all SBPs including fragmentary pieces, the variation in the dimension of preforms is larger than that of SBPs and shows a greater scatter even for thickness which is not included in the scatterplot (Fig 10).

The split, which is crucial for these Aurignacian antler points, shows variation. The split created for hafting is visible even when the proximal ends are not present. Some show that the force propagated through the long axis resulting in a fissure that leads to the uneven stepped fracture, which at times is accompanied by the exposure of the inner face of the split. Some split breaks are visible even on distal tips of small dimensions (Fig 2.3). These small SBPs, as well as other fragmentary points, show that the pieces were exposed to longitudinal pressure. Furthermore, we observe examples of failed attempts at splitting the point, resulting in a split running on the cortical face and not on the lateral sides. One SBP exhibits three splits, one on both sides in addition to one of the cortical faces.

By measuring the distance between the beginning of the split to the distal point, we observe that the split runs 5 to 62% of the entire length of SBPs when the distal tip is preserved or nearly complete. One piece exhibits a split that is merely 26.7 mm away from the distal tip (Fig 2.14). This piece is further characterized by an overall small dimension and resharpening of the distal tip at an angle, which gives it an asymmetrical profile when viewed from the lateral side. The small dimensions of such pieces can be attributed to the maintenance and reuse of SBP. This example in addition to other SBPs with smaller dimensions indicates break after use as well as the curation of points for maintenance and reuse.

## Discussions

### Spatial and temporal contexts

The sites in the Swabian Jura have yielded one of the highest concentrations of SBP outside of the Aquitaine Basin and Cantabria, which are other hotspots of the Aurignacian culture. SBPs existed at many sites in the Swabian Jura where the Aurignacian record is rich. The exceptions are Hohlenstein-Stadel and Sirgenstein. Both sites have produced possible preforms or possible MBPs. The general abundance shows that SBP occurred more frequently than MBP. Antler MBPs possibly existed in the region, but no worked remain demonstrates a rounded proximal end which we can assign to this type. This indicates an ephemeral presence of antler MBP in the Aurignacian of the Swabian Jura and reflects a technological affinity to sites in Western Europe as opposed to Eastern Europe. An in-depth comparison of MBPs may provide further insight into the variation and possible parameters useful in the identification of MBPs [39].

The stratigraphic contexts of SBPs demonstrate that they are present in the earlier horizons of the Swabian Aurignacian. This is true for Hohle Fels and possibly for Vogelherd. While horizons of Vogelherd are not securely dated and analyzed in a systematic manner which is comparable to Hohle Fels and Geißenklösterle, Riek’s documentation suggests that the SBPs were more abundant in the lower horizons (V) (1934). The contrary is true for Geißenklösterle, which has been previously cited as evidence of a relatively late occurrence of the ‘Early Aurignacian’ in this region [101, 102]. This paper demonstrates that intersite variability can account for the absence of SBP at Geißenklösterle regardless of the early AMS dates [90]. Furthermore, the occurrence of SBPs from different stratigraphic contexts highlights how cross-examining patterns across sites is vital for obtaining regional signals and exploring the complexity of the Aurignacian on a broader scale.

### Operational sequence

**Fig 11:**
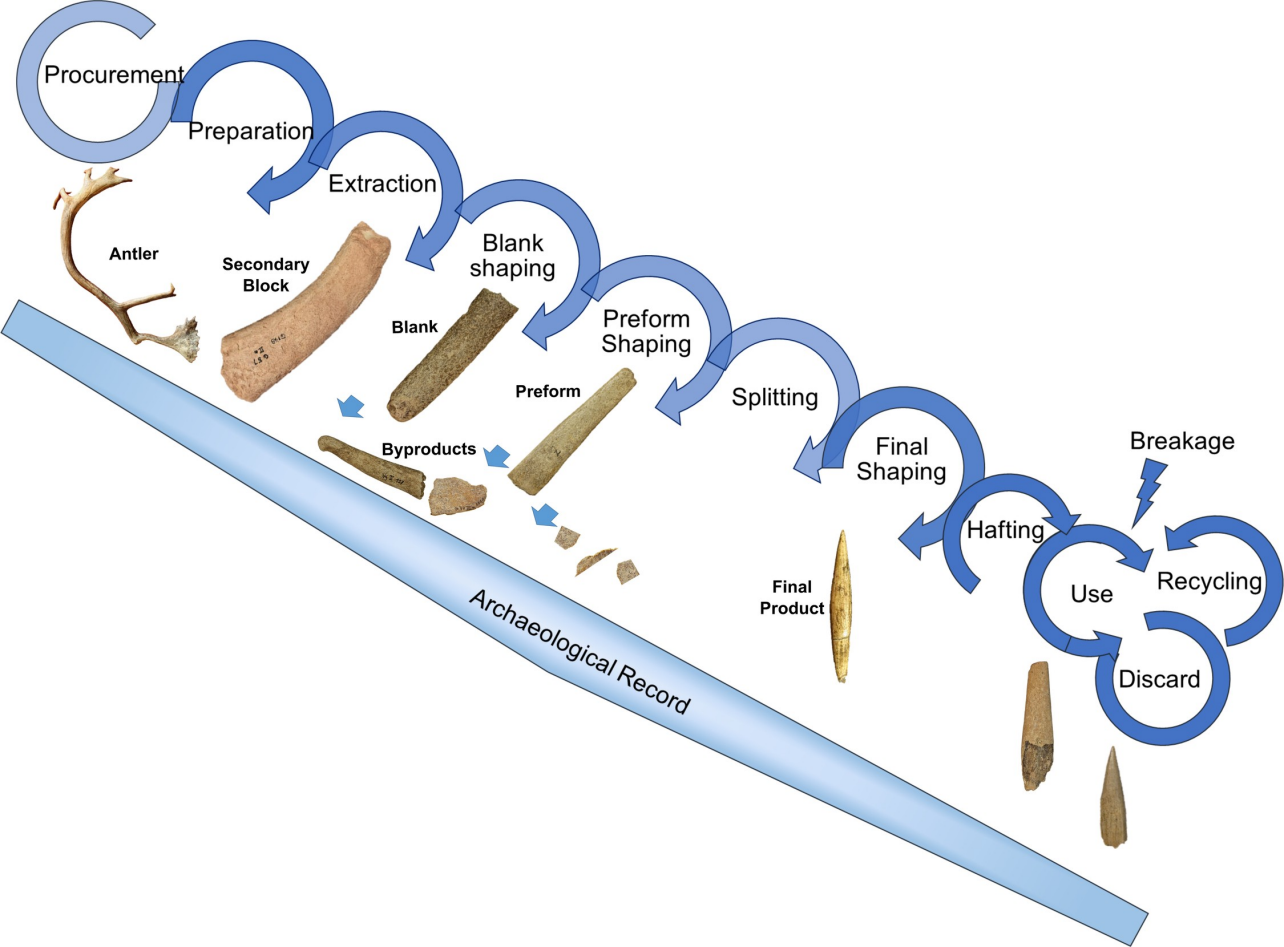
Schematic representation of the operational sequence of SBPs in the Swabian Jura * artifacts not to scale.

As summarized in Fig 11, the techniques involved in the SBPs resemble that of other regions [44, 45, 49]. There are subtle differences that merit additional discussion.

#### Blank production

Upon studying the blank production of SBP from Castanet, Tartar concluded that makers often used direct percussion, which was evidenced by notches and impact fractures on the lateral edges of the artifacts [103]. This does not necessarily exclude the use of other methods. On the contrary, indirect percussion with the use of wedge is proposed by several authors [42, 45, 49]. Splitting and wedging or indirect percussion often does not leave clear technological traces and is difficult to identify on the surfaces of antlers. Its use is demonstrated with one blank at the Swabian Jura, again leaving ample room for multiple extraction methods. Experiments have adopted the use of wedges for the initial extraction of blanks due to the greater level of control [44, 51]. Their studies highlight flexibility in the methods adopted by the makers and the possible absence of standardization that is at times assumed in technological studies at this stage of the tool production.

#### Preforms

The dimension of preforms overlaps generally with that of the complete SBPs. Since the length of preforms and SBPs is generally affected by breakage through manufacture, use or post-depositional processes, this factor may not be relevant to the production of antler points. Conversely, the width and thickness are less influenced by use and/or taphonomic processes as fractures on SBPs disproportionally affect the distal pointed ends, which are more fragile. The overlap in the values of thickness indicates that pieces are close to completion before the split. The removal of the cancellous material on the preform can explain some variation in the width.

The difference in width is significant and highlights the reduction of the proximal base through shaping the lateral edges. This invokes two possible explanations. The splitting technique through the use of incision and flexion would have resulted in the narrowing of the base due to the removal of the base from the preform, whose form is often triangular. An alternative explanation posits that the split using cleavage was either preceded or followed by an additional narrowing of the base. If this step preceded the split, it may have facilitated the action of splitting or if it followed the split, it was likely informed by the dimension of the shaft that was later attached to the SBP for its use as a projectile weapon. In any case, the difference in the width of preforms and complete SBPs merits future study to explore the splitting method and other technological variations in the SBP production.

#### Splitting method

No clear tongued pieces, such as those securely associated with SBPs in other regions, have been identified in the Swabian Jura. This merits revisiting the discussion on the splitting method. The simple method of splitting through the use of cleavage has been proposed and demonstrated by Henri-Martin (1931) [48]. This interpretation has been supported by observations of several studies, showing that there is little to no loss of material between the proximal wings [43, 48]. Peyrony 1928 first proposed the method of incision and flexion, which results in tongued pieces. A method involving the use of wedges may have been more frequently employed than that of the incision and flexion technique, although the latter appears to be easier to perform [44].

In our study, we cannot positively verify the use of incision and flexion technique. Possible explanations include:

1. The use of the incision and flexion technique was rare in the region [42, 49]. Instead, the makers produced splits in an alternative method with the application of cleavage or wedge.
2. Tongued pieces were recycled, given that some pieces can relatively be large in dimension [103] and could have been reworked through shaping for other functions.
3. The final stages of the SBP production including splitting were completed outside of the base camps, perhaps close to the hunting locales.

These explanations are not mutually exclusive. The lack of tongued pieces has been documented elsewhere, notably in Belgium and Eastern Europe. The most recent study exploring this topic indicates that both methods were possibly in use [44]. Incision and flexion can initiate the split and afterward, the makers can employ cleavage or wedge to extend the split. If the absence of tongued pieces remains an archaeological reality in regions outside of the Aquitaine Basin and Cantabria, researchers would need to explain the potential variation in the splitting method. Suffice to say that future excavation and new analyses will help explore these possible competing hypotheses.

#### Size, form and split

Variation in size and forms has been attested from previous studies with a relatively large sample [40, 41, 43]. The lack of standardization also adds weight to the hypothesis that makers often engaged in reuse and maintenance of SBPs [45, 49]. This has implications for understanding the category of forms. Through the curation process, one form can likely change into another. Thus, forms should be interpreted as a fluid category which can alter throughout the life history of points. The recurrent use of artifacts enables us to explore the intensity of their use. In the future, resharpening can be explored when more quantitative data on the split, including the distance between the distal tip and the split, become available for a greater number of assemblages.

The average dimensions of SBPs from the Swabian Jura are significantly smaller compared to SBPs from other European sites. In particular, Vogelherd has produced the largest assemblage of SBPs and drives the general pattern in the Swabian Jura. The pattern is a possible artifact that can be tied to the recovery technique. Some large assemblages stem from older excavations without systematic sieving which would enable the retrieval of smaller material. This means that the majority derives from modern excavations with extensive sorting, which can result in the recovery of smaller points. The alternative scenario is that more intensive use of SBPs led to more frequent breakage and smaller dimensions of SBPs. These hypotheses can be tested when a larger body of work from recent excavations outside of the Swabian Jura is available for comparative analysis.

The small dimension also points to the possibility of additional hafted material, which may have been critical for its effectiveness as a handheld or throwing projectile armature. Hahn previously proposed the possible attachment of bladelets on the shaft [40]. The combination of osseous and lithic components would allow the makers to take advantage of both materials for effective hunting: the latter for piercing through the skin of hunted animals and the former for extending the sustained damage and bleeding [104]. While experimental work will allow us to test this possibility, the likelihood of the SBP as a composite tool remains plausible due to the small dimension of the Swabian SBPs. This being said, we have yet to identify evidence from lithics such as the traces of mastic that would support this hypothesis.

#### Reflecting on antler technology

The SBPs mark the formal tool making combined with the exploitation of new raw material. Antlers were resources that were readily available on the landscape but remained untapped for most of the timespan that hominins evolved in Europe and western Asia. They differ from lithic raw materials in that their distribution reflects that of reindeer, which increased in parts of Western and Central Europe during the eUP [75, 105, 106]. The incorporation of antlers meant a greater investment of time and energy in the production and longer use of the manufactured points. This suggests a diversified approach to preparation, maintenance and use of tools, which differ from that of lithics.

Modern humans occupied ecological niches with greater breadth than that of their predecessors by exploiting an array of raw material, which was previously untapped by hunter-gatherers, including Neanderthals who largely practiced osseous technology based on expedient tools with few exceptions [21, 65, 107, 108]. The exploitation of antlers by modern humans speaks to the technological innovation not just in forms and techniques, but also reflects the incorporation of new resources within the technological systems of the Aurignacian. The use of antlers added diversity to the technological repertoire, reflecting the adaptability of modern humans.

## Conclusions

The analyses of organic tools have the potential to address the multifaceted aspects of the economic, social and cultural activities of humans. The overview of SBPs in the Swabian Jura, the largest in Central Europe, reveals inter-regional parallels and variability. In terms of techniques, the assemblages lack tongued pieces despite the abundance of SBPs. One of the hypotheses is that the alternative splitting method was more commonly employed. Additionally, the difference in the maximum width between the preforms and finished points indicates that the reduction of the proximal base resulted from the splitting method or was a crucial step in the production of projectile points. SBPs are also a unique Aurignacian phenomenon and have implications for understanding the technocomplex. Assemblages from Geißenklösterle, Hohle Fels, and Vogelherd comprise the majority of the antler points. In the assemblages, SBPs are far more common than MBPs made from antlers, a pattern that suggests a stronger cultural link to the Early Aurignacian of western Europe. The diversification of tool type, raw material, and technique continue to provide insights into the behavioral innovation of modern humans, which is exemplified by the early Upper Paleolithic record of Europe.

## Supporting information

S1 File Complete split-based points and fragments of split-based points with their dimensions and locations. (PDF)

S2 File Preforms of split-based points and their measurements with their dimensions and locations. (PDF)
