# Supplementary material for: Split-based points from the Swabian Jura highlight Aurignacian regional signatures
Source: PLoS One. 2020 Nov 10;15(11):e0239865. doi: 10.1371/journal.pone.0239865 (PMC7654757; doi:10.1371/journal.pone.0239865)
Supplement: S1 File — (PDF) [file pone.0239865.s001.pdf]

Site	Square	ID Number	AH	portion	length (mm)	width (mm)	thickness (mm)	Location
Bockstein Höhle		9		complete	147.9	22.5	5.9	Ulmer Museum
Brillenhöhle		V56,9-2	XIV	almost complete	87.9	22.05	9.01	Landesmuseum Württemberg
Geißenklösterle	15	236	IIa	mesial-proximal	34.78	10.46	6.54	University of Tübingen
Geißenklösterle	25	248	IIb	mesial-proximal	51.71	10.3	6.56	Landesmuseum Württemberg
Geißenklösterle	27	4	IIc	mesial-proximal	36.87	7.68	5.17	Landesmuseum Württemberg
Geißenklösterle	36	334	IIb	complete	77.82	6.56	4.92	University of Tübingen
Geißenklösterle	37	129	IIa	mesial-proximal	44.74	10.6	5.4	University of Tübingen
Geißenklösterle	46	39	IIb	proximal wing	29.14	10	3.16	University of Tübingen
Geißenklösterle	56	120	IIb	almost complete	66.32	9.33	6.57	Landesmuseum Württemberg
Geißenklösterle	57	578	IIb	proximal wing	14.97	8.03	2.46	University of Tübingen
Geißenklösterle	68	162	IIb	complete	114.85	7.6	6.69	Landesmuseum Württemberg
Geißenklösterle	68	332	IIab	distal	19.5	5.2	3.8	University of Tübingen
Geißenklösterle	68	481	IIb	almost complete	46.5	9.03	3.35	University of Tübingen
Hohle Fels	30	1120	Va	proximal wing	27.28	8.77	2.79	University of Tübingen
Hohle Fels	30	1210	Vb	complete	52.08	4.65	3.3	URMU
Hohle Fels	31	2616	Vaa	proximal wing	23.85	8.71	2.83	University of Tübingen
Hohle Fels	32	2172	Vabwf	almost complete	57.01	11.02	6.6	University of Tübingen
Hohle Fels	66	4332.1	IV	mesial-proximal	40.79	11.82	3.66	University of Tübingen
Hohle Fels	67	2843	IV	mesial	45.18	10.14	3.74	University of Tübingen
Hohle Fels	68	2784	IV	proximal wing	28.93	8.4	1.94	University of Tübingen
Hohle Fels	75	628.2	IVwf	mesial	35.67	10.35	4.48	University of Tübingen
Hohle Fels	76	1486	IV	distal	46.45	7.8	3.7	University of Tübingen
Schafstall II		20	AUR	mesial-proximal	58.1	13	7.4	University of Tübingen (temp)
Vogelherd		2	V	complete	81.5	9.2	6.7	MUT
Vogelherd		16	IV	proximal wing	23.1	9.5	2.9	University of Tübingen
Vogelherd		67	V	distal	35.8	6.9	4.8	University of Tübingen
Vogelherd		68	V	distal	37.7	7.7	4.8	University of Tübingen
Vogelherd		85.2	V	distal	43.9	7.3	3.8	University of Tübingen
Vogelherd		86.2	V	distal-mesial	36.9	6.8	4.8	University of Tübingen
Vogelherd		87.1	V	almost complete	57.3	7.8	3.8	University of Tübingen
Vogelherd		104	V	complete	63.6	9.8	5.6	University of Tübingen
Vogelherd		125.3	V	complete	53.6	8.4	6	University of Tübingen
Vogelherd		125.1	V	complete	76.6	12.1	6.7	University of Tübingen
Vogelherd		125.2	V	complete	61.8	8.5	5.6	University of Tübingen
Vogelherd		126.1	V	almost complete	54.7	12.3	7	University of Tübingen
Vogelherd		127.5	V	complete	38.6	7.5	5.3	University of Tübingen
Vogelherd		128	V	complete	51.8	9.7	6.8	University of Tübingen
Vogelherd		211		distal	43.5	9.9	6.4	University of Tübingen
Vogelherd		212		mesial-proximal	40	8.6	5.4	University of Tübingen
Vogelherd		249	V	complete	84.1	9.4	7.3	MUT
Vogelherd		A3384-23	V	almost complete	72.3	7.87	6.15	Landesmuseum Württemberg
Vogelherd		A38/130-2		distal-mesial	45.12	8.94	5.1	Landesmuseum Württemberg
Vogelherd		V72,39-2		distal-mesial	33.4	6.65	4.06	Landesmuseum Württemberg
Vogelherd		V72,39-3		distal-mesial	41.84	7.42	4.58	Landesmuseum Württemberg
Vogelherd		V72,39-4		distal	26.13	5.83	4.54	Landesmuseum Württemberg
Vogelherd	33/71	51.1		complete	47	6.5	5.2	University of Tübingen

Vogelherd	34/70	15.1		mesial-proximal	33.8	9.9	5.5	University of Tübingen
Vogelherd	34/76	24.1		proximal wing	18.7	8.5	3.6	University of Tübingen
Vogelherd	35/71	47.1		proximal wing	23.7	6.9	2.7	University of Tübingen
Vogelherd	35/77	82.1		mesial	35.7	11.5	5.3	University of Tübingen
Vogelherd	36/76	22.1		distal-mesial	29.8	7.7	6	University of Tübingen
Vogelherd	36/77	44.1		mesial-proximal	43.8	11	5.6	University of Tübingen
Vogelherd	37/71	25.1		proximal wing	22.3	10	2.9	University of Tübingen
Vogelherd	37/72	99.1		almost complete	47.5	9.9	7.4	University of Tübingen
Vogelherd	37/74	123.1		almost complete	51.4	10	5.7	URMU
Vogelherd	37/77	80.1		mesial-proximal	39.2	8.6	4.7	University of Tübingen
Vogelherd	38/67	88		almost complete	55.33	9.9	6.1	University of Tübingen
Vogelherd	38/74	30.1		almost complete	37.5	9	5.6	University of Tübingen
Vogelherd	38/75	114.1		proximal wing	20.2	6.5	2.7	University of Tübingen
Vogelherd	38/77	53.2		complete	37.1	6.7	3.8	University of Tübingen
Vogelherd	39/67	254.1		mesial wing	25.8	5.9	2.3	University of Tübingen
Vogelherd	39/76	41.1		mesial	32.9	8.1	6.3	University of Tübingen
Vogelherd	40/69	35.1		mesial	24.4	7.9	3.9	University of Tübingen
Vogelherd	40/71	54		complete	52.1	8.3	5.8	University of Tübingen
Vogelherd	40/71	66.1		distal	43	6.7	4.3	University of Tübingen
Vogelherd	40/71	71.1		distal-mesial	32.7	7.8	4.7	University of Tübingen
Vogelherd	40/72	41		almost complete	55.3	9.8	6.3	URMU
Vogelherd	41/69	121.2		mesial wing	23.3	9	3.6	University of Tübingen
Vogelherd	42/61	43.1		distal	24.5	7.1	5.5	University of Tübingen
Vogelherd	42/70	96.1		almost complete	49.5	10.2	5.4	University of Tübingen
Vogelherd	43/69	43.2		proximal wing	20.3	7.6	2.2	University of Tübingen
Vogelherd	43/70	19		almost complete	49	5.8	4.1	University of Tübingen
Vogelherd	44/67	25		distal	20.7	6.7	2.6	University of Tübingen
Vogelherd	44/68	95.1		distal	33	7.4	4.7	University of Tübingen
Vogelherd	46/64	30.2		mesial-proximal	37.24	11.4	6.5	University of Tübingen
Vogelherd	56/65	28.1		mesial	27.2	9.7	5.4	University of Tübingen
Vogelherd	60/63	40.1		distal-mesial	22.8	5.4	3.8	University of Tübingen
Vogelherd	62/60	20.1		distal	49	8.4	4.8	University of Tübingen
Vogelherd	62/61	30.2		proximal wing	20.5	7.9	2.1	University of Tübingen
Vogelherd	63/62	31		complete	48.2	9.9	4.7	University of Tübingen
Vogelherd	65/61	72		mesial-proximal	50.7	10.5	6.8	University of Tübingen
Vogelherd	65/64	73.1		distal	30.8	6.5	3.7	University of Tübingen
Vogelherd	67/62	19		proximal	27.6	9.8	3.1	University of Tübingen
Vogelherd	67/63	10.1		complete	37	8.8	4.6	University of Tübingen
Vogelherd	73/63	29.1		complete	43.6	8.7	5.8	University of Tübingen
Vogelherd	81/63	92.1		distal	19.2	5.9	4.8	University of Tübingen
Vogelherd	83/65	25		almost complete	63.9	8.23	4.75	University of Tübingen
Complete and fragments of split based points								
