# Supplementary material for: Split-based points from the Swabian Jura highlight Aurignacian regional signatures
Source: PLoS One. 2020 Nov 10;15(11):e0239865. doi: 10.1371/journal.pone.0239865 (PMC7654757; doi:10.1371/journal.pone.0239865)
Supplement: S2 File — (PDF) [file pone.0239865.s002.pdf]

Site	Square	ID Number	AH	portion	length (mm)	width (mm)	thickness (mm)	Location
Bockstein Höhle		Z		complete	100.5	23.7	7.1	Ulmer Museum
Bockstein Törle		4619	VII	complete	117.8	18.4	5.95	Ulmer Museum
Bockstein Törle		6.9.33-1		complete	94.15	24.43	8.46	Ulmer Museum
Brillenhöhle		220	XIV	proximal	55.44	24.27	6.36	Landesmuseum Württemberg
Hohle Fels	28	1851.1	Vaa	mesial	25.4	8	5	University of Tübingen
Hohle Fels	99	1938	IV	complete	92.1	27.99	12.02	University of Tübingen
Hohlenstein-Stadel		6H.1	5/6H	half	101.3	17.07	6.78	Ulmer Museum
Sirgenstein		SG 1	V	complete	173	15	8.7	University of Tübingen
Vogelherd	34/70	54.1		mesial	28.7	10.2	5	University of Tübingen
Vogelherd	38/73	127		complete	92.47	20.69	5.95	University of Tübingen
Vogelherd	40/67	111.1		mesial	28.6	12	6.4	University of Tübingen
Vogelherd	42/70	23		mesial	17.9	10.8	5.3	University of Tübingen
Vogelherd	43/70	91		mesial	40.6	10.7	5	University of Tübingen
Vogelherd	44/70	80		mesial	43.8	11.6	6	University of Tübingen
Vogelherd	44/71	69.1		mesial	42.6	9.8	5.6	University of Tübingen
Vogelherd	45/67	73		mesial	37.2	10.7	4.8	University of Tübingen
Vogelherd	46/67	11.1		mesial	29.8	9.6	5.3	University of Tübingen
Vogelherd	58/64	7		mesial	30	9	4	University of Tübingen
Vogelherd	62/63	31.1		mesial	29	9.3	4.6	University of Tübingen
Vogelherd	65/65	56		mesial	31.8	8	5	University of Tübingen
Vogelherd		15	IV	mesial	47.6	15.4	7	University of Tübingen
Vogelherd		65	V	complete	69.8	9	4.6	University of Tübingen
Vogelherd		22	IV?	complete	79.1	19	8.9	University of Tübingen
Vogelherd		71	V	complete	75.4	14.6	10	University of Tübingen
Vogelherd		125.1	V	complete	76.6	12.1	6.7	University of Tübingen
Vogelherd		3.2 (1)	IV	complete	56.8	12	6.4	University of Tübingen

Preforms of split based points and their measurements

(1) discussed as possible MBP in the text as well
